# Arabinoxylan-Based Microcapsules Being Loaded with Bee Products as Bioactive Food Components Are Able to Modulate the Cell Migration and Inflammatory Response—In Vitro Study

**DOI:** 10.3390/nu14122529

**Published:** 2022-06-17

**Authors:** Gabriela Kowalska, Justyna Rosicka-Kaczmarek, Karolina Miśkiewicz, Małgorzata Zakłos-Szyda, Sascha Rohn, Clemens Kanzler, Magdalena Wiktorska, Jolanta Niewiarowska

**Affiliations:** 1 Institute of Food Technology and Analysis, Faculty of Biotechnology and Food Sciences, Lodz University of Technology, 90-537 Lodz, Poland; gabriela.kowalska@p.lodz.pl (G.K.); karolina.miskiewicz@p.lodz.pl (K.M.); 2 Institute of Molecular and Industrial Biotechnology, Lodz University of Technology, 90-537 Lodz, Poland; malgorzata.zaklos-szyda@p.lodz.pl; 3 Department of Food Chemistry and Analysis, Institute of Food Technology and Food Chemistry, Technische Universität Berlin, 13355 Berlin, Germany; rohn@tu-berlin.de (S.R.); clemens.kanzler@tu-berlin.de (C.K.); 4 Department of Molecular Cell Mechanisms, Medical University of Lodz, 92-215 Lodz, Poland; magdalena.wiktorska@umed.lodz.pl (M.W.); jolanta.niewiarowska@umed.lodz.pl (J.N.)

**Keywords:** rye bran, bioactive heteropolysaccharides, arabinoxylans-based microcapsules, bee products, biostability, inflammatory response

## Abstract

The aim of the research was to use bioactive heteropolysaccharides isolated from rye bran to obtain innovative systems for the controlled release of bioactive compounds. The core of the obtained encapsulates was honey and royal jelly. It was shown for the first time that preparations effectively ameliorated inflammatory response in lipopolysaccharide (LPS)-treated RAW 264.7 macrophages, decreasing the secretion of interleukin 6 (IL-6), tumor necrosis factor α (TNF-α) and nitric oxide (NO). The in vitro digestion process revealed that bee products’ encapsulates were stronger oxidative stress reducers and had sustained ability to reduction in inflammation state mediators. The lack of inhibitory effect on migration rate of human microvascular endothelial cells (HMEC-1) endothelial cells and mouse embryonic fibroblasts (NIH-3T3), both cell models involved in wound healing process, additionally identified these preparations as agents potentially used in the management of inflammatory response. In the process of a simulated digestion in vitro, the innovative microcapsules showed 85% higher biostability and two to ten times better bioavailability, compared to natural bee products.

## 1. Introduction

Although being relevant food products, royal jelly and honey are known for their therapeutic and prophylactic properties, which are based mainly on their antibacterial, anti-inflammatory, and antioxidant activities [1,2,3,4]. Several data indicated that application of honey or royal jelly reduces the secretion of inflammatory mediators such as interleukin 1 (IL-1), interleukin 6 (IL-6), tumor necrosis factor α (TNF-α), and nitric oxide (NO), as well prostaglandins [2,5]. These exert an anti-inflammatory effect not only by scavenging of free radicals and reactive oxygen species (ROS), but also by involving other anti-inflammatory factors such as major royal jelly protein (MRJP) and medium-chain fatty acids, such as 10-hydroxydecenoic acid. In vivo and in vitro studies showed that MRJP-3 has potent immunomodulatory activity inhibiting the production of interleukin 4 and 2, and interferon-γ (IFN-γ) by T-cells associated with suppression of cell proliferation [6]. Chen et al. analyzed the anti-inflammatory effects of the main three fatty acids present in royal jelly: trans-10-hydroxy-2-decenoic acid (10-H2DA), 10-hydroxydecenoic acid (10-HDA), and sebacic acid (SEA) [7]. The results of the studies showed that 10-H2DA, 10-HDA and SEA had a strong dose-dependent inhibitory effect on the release of NO and interleukin 10 in mice LPS-stimulated macrophages RAW 264.7, while only SEA reduced the concentration of TNF-α. This effect can be also matched with the high levels of bioactive compounds present in bee products such as enzymes, flavonoids, phenolic acids, organic acids, carotenoids, ascorbic acids, and amino acids, but Maillard reaction products also contribute to the hypothesized antioxidant and anti-inflammatory [4].

Numerous literature reports that bee products, i.e., honey or royal jelly may, depending on various plant sources, increase or reduce skin cell migration in wound healing processes, e.g., by preventing the migration of neoplastic cells [8,9]. It is still largely unknown whether different types of royal jelly and honey have different potency and which are the bioactive ingredients and mechanisms underlying their effectiveness. It is known that disturbances in the natural cascade of wound healing processes (proliferation, migration and differentiation of fibroblasts and keratinocytes) can lead to the formation of difficult-to-heal wounds and scars. Therefore, it is important to look for such preparations, which would be bioavailable to cells, support the natural healing processes by immunomodulating and antibacterial activity, and at the same time do not uncontrollably enhance the migration and differentiation processes leading to scarring and fibrosis.

The anti-inflammatory and antioxidant activities of phenolic compounds are well known and have been confirmed by numerous in vitro studies. However, still many conflicting results regarding bioactivity of phenolic compound under in vivo condition are discussed. This disagreement is related to the phenolic compounds metabolism in the gastrointestinal tract [10], as the effectiveness in disease prevention by dietary agents depends on preserving the bioavailability of the active ingredients. Due to insufficient gastric residence (variable pH, different enzymes’ activities), solubility and/or permeability within the gut, as well as the lability of bioactive compounds to processing conditions such as temperature, oxygen, light, or interaction with the food matrix, only the part of phenolic compounds remain available [11,12,13]. Maintaining the biological activity of health-promoting components can be achieved by including labile materials in capsules [13]. With regard to recent trends in innovative food technology, special attention is paid to encapsulation. In the food industry, the microencapsulation process protects the core bioactive material from its degradation by reduction in its reactivity, adjusting of the location and time of its release, optimization of the physical properties to enable easier handling and maintaining the active molecular form until the time of consumption, as well as its delivery to the physiological target within the organism. In connection with the above, the application of encapsulated bioactive compounds could enable the extension of the scale of their utilization [14].

Given the limited biostability and bioavailability of bioactive compounds present in bee products, obtaining a controlled delivery system in the form of microcapsules could overcome some restraints. Microcapsulation of bioactive compounds can be achieved by means of spray drying, which is one of the most commonly used encapsulation methods in the food industry, due to its relatively low cost, high availability of equipment. More important, the low heat load of the material enables its application for thermolabile substances [15]. As coating substances modified starch, maltodextrin, gum arabic, chitosan, or cyclodextrin are predominantly used. The wall material must be soluble in water and its molecular mass should lead to an increase in glass transition temperature, which determines the encapsulation process. So far, several works have been published in which carriers such as maltodextrin, gum arabic, or nutriose were used for honey encapsulation by spray drying method with feed solids minimal amount about 20–50% depending on carrier type [16,17,18]. It is worth noting that apart from nutriose, exhibiting prebiotic properties, the commonly used coating materials (maltodextrin, gum arabic, etc.) have low nutritional value. Obviously, encapsulation of valuable products such as honey and royal jelly should be performed by the use of carriers with health-promoting properties with additional biological value. Natural heteropolysaccharide (HPS)-based delivery systems may be the optimal choice, as they are biocompatible, biodegradable, and have a wide range of functional properties. Moreover, HPS are suitable for entrapment of both hydrophilic as well as hydrophobic compounds by possible interaction with their functional groups. HPS have high temperature stability, and due to this advantage, they can be used as a wall material for spray drying encapsulation process instead of labile protein- or lipid-based delivery systems [19]. More recently, water-extractable arabinoxylans (WEAX) have been studied for the development of HPS-based delivery systems [20,21,22,23,24]. WEAX are hemicelluloses composed of a β-(1–4)-xylopyranose linear backbone chain with α-L-arabinose substitution at O-3 and/or O-2 position. Arabinose can be esterified with ferulic acid (FA) molecules at O-5 position [25]. This characteristic enables formation of gels by covalent crosslinking of FA molecules. The crosslinking of arabinoxylans (AX) chains leads to the formation of dimers (di-FA) and trimers (tri-FA) of FA, resulting in the three-dimensional network of the gel [26,27]. Such crosslinked WEAX gels are suitable as matrices used for controlled release delivery systems of bioactive ingredients, but also this polysaccharide matrix can reveal other significant functional properties. As demonstrated by Kowalska et al., encapsulation of honey with rye bran WEAX entrapment resulted in an increase in antioxidant activity and total phenolic compounds content (TPC) of honey-loaded microcapsules in comparison to native honey [24]. This result seems to be even more significant in the light of other rye bran WEAX activities beneficially affecting human health by exhibition of anti-inflammatory and anti-cancer properties, prevention of constipation, blood cholesterol lowering, regulation of blood glucose level, and enhancement of the minerals absorption. As in the large intestine WEAX can be fermented to short chain fatty acids (SCFA) with prebiotic properties stimulating the growth of lactic acid bacteria, WEAX are suitable for the development of colon-specific delivery systems [28].

Consequently, the aim of the present study was to determine the properties of bioactive compounds contained in WEAX-based microcapsules loaded with honey or royal jelly in regard to the inflammation and cell migration. In addition, the effect of an in vitro simulated digestion process in the gastrointestinal tract on the antioxidant capacity and the bioavailability of fixed bee products has been checked. In experiments based on modulation of cell migration, human microvascular endothelial cells HMEC-1 and murine embryonic fibroblasts NIH-3T3 were used, whereas the inflammatory response was investigated using murine macrophages RAW 264.7 cell line. The chosen types of cell lines resemble the cells involved in regulation of inflammatory response.

## 2. Materials and Methods

### 2.1. Materials

Among heteropolysaccharides, WEAX were isolated from rye bran, a by-product of the milling industry (Szadek Mill, Szadek, Poland). The rye bran (wholegrain) was subjected to water extraction at 20 °C for 2 h, according to a previously developed method [27]. Both, honeydew honey (HH) and royal jelly (RJ) was sourced from a local apiary (Ekoostoja, Poland). Elementary physicochemical parameters of HH and RJ were as follows: water content 18.7 and 63%, water activity 0.538 and 0.837, protein content 0.71 and 10.4%, glucose content 39.3 and 10.8 g 100 g^−1^ and fructose content 41.6 and 11.2 g 100 g^−1^, respectively. Potassium nitrosodisulfonate (Frémy’s salt), 2,2-diphenyl-1-picrylhydrazyl radical (DPPH), copper sulfate pentahydrate, 6-hydroxy-2,5,7,8-tetramethylchroman-2-carboxylic acid (Trolox), dipotassium phosphate, monopotassium phosphate, pancreatin from porcine pancrease (207 protease units per mg of solid mass; 238 α-amylase units per mg of solid mass; and 29.9 lipase units per mg of solid mass), pepsin from porcine gastric mucosa (474 units per mg of solid mass), sodium carbonate, phenolic standards, HPLC-grade methanol, acetonitrile and HPLC-grade formic acid were purchased from Sigma Aldrich Co. LLC (St. Louis, MO, USA).

### 2.2. Preparation of Honey-Loaded Microcapsules

The encapsulation process was carried out according to our own method described in Kowalska et al., Briefly, the WEAX with a concentration of 5% dry basis was stirred for 1 h at 20 °C [24]. The solution was then stirred for 1 h at room temperature (20 °C). Next, 10 g of HH or 50 g of RJ was added to WEAX solution. The ratio of core material to the carrier was 1:4 and 1:20 for HH and RJ encapsulates, respectively. The mixtures were stirred on a magnetic stirrer for 22 h at room temperature (20 °C), in order to crosslink the arabinoxylans fraction. The obtained mixtures were spray dried to produce microcapsules of honey (HH-WEAX) or royal jelly (RJ-WEAX). The drying process was carried out using a Mini Spray Dryer B 290 (Büchi Labortechnik GmbH, Essen, Germany).

### 2.3. Antioxidant Properties

#### 2.3.1. Preparation of Extracts

In order to determine the antioxidant activity of the formulation of fixed bee products (HH-WEAX and RJ-WEAX), non-encapsulated honey, non-encapsulated royal jelly, and WEAX rye bran, the 50% aqueous acetone extracts were prepared with a sample-to-solvent ratio of 1:10 (*w*/*v*) and extracted for 24 h at room temperature (20 °C). 

#### 2.3.2. Determination of Antioxidant Activity by Electron Paramagnetic Resonance (EPR) Spectroscopy

The EPR spectroscopic measurements were performed with the stabilized radicals of Frémy’s salt and DPPH. Aliquots (100 μL) of the water/acetone extracts were allowed to react with an equal volume of an aqueous 2 mmol solution of Frémy’s salt. For determination of the antioxidant activity with DPPH, aliquots of extracts (100 μL) were mixed with an equal volume of 1 mmol ethanolic DPPH solution. A Trolox standard (0.25 mM) was used as reference. For determination of the antioxidant activity the relative radical concentration was determined after 10 and 30 min for Frémy’s salt- and DPPH-assay, respectively. Spectra were measured on a Miniscope MS 100 spectrometer (Magnetech GmbH, Berlin, Germany). Microwave attenuation for both measurements with stabilized radicals was set at 10 dB. Modulation amplitude was set at 0.15 and 0.10 mT for Frémy’s salt and DPPH, respectively, and magnetic flux density was set at 338.8 and 339.5 mT, respectively. The 30 sweep wide was set at 70 and 100 G for Frémy’s salt and DPPH, respectively, and sweep time was set at 30 s.

#### 2.3.3. Determination of Cu^2+^-Ion Chelating Capacity by EPR Spectroscopy

Complex solutions were prepared by mixing 750 μL extract with the 250 μL 10 mM copper (II) solution. Then, the mixture of 250 μL complex solution with 750 μL buffer (50 mM phosphate buffer, pH 7.4) or water was transferred into an EPR capillary [29]. Spectra were measured on a Miniscope MS 100 spectrometer (Magnetech, Berlin, Germany). Microwave attenuation and modulation amplitude was set at 10 dB and 10.0 mT, respectively. Centre field was set at 312.0 mT and sweep time at 30 s. Every sample was measured 16 times and the spectra were averaged. In addition, a blank (water) and a copper (II) solution without ligand were measured each time to correct the spectra [30].

### 2.4. Simulated Gastrointestinal Digestion

A static in vitro digestion model comprising two-stages of digestion (gastric and intestinal) as proposed by Minekus et al., with some modifications was applied in HH, RJ, WEAX, and microcapsules (HH-WEAX, RJ-WEAX) [31]. Briefly, the protocol based on the use of 2 simulating fluids, gastric juice at pH 2 (SGF) and intestinal juice at pH 7 (SIF). Then, 200 mg of the preparations were weighed into the 50 mL sterile centrifuge tubes and mixed with 14 mL 0.1 M HCl. The mixture was acidified to pH 2.0 by the addition of 6 M HCl solution and treated with pepsin (6000 units). After the solutions were thoroughly mixed, the tubes were incubated in a shaking water bath at 37 °C for 2 h. The pH was subsequently adjusted to 7 using 0.5 M sodium bicarbonate. Then, 15 mL of SGF were collected and placed in 50 mL sterile centrifuge tubes. Solutions were centrifuged (3024× *g*, 60 min, 4 °C). The supernatants were decanted and transferred to clean 50 mL sterile centrifuge tubes and freeze-dried. For digestion with SIF, 1 mL of pancreatin solution (100 mg of pancreatin in 20 mL of 0.1 M NaHCO_3_) and 1 mL of bile salt solution (500 mg of bile salt dissolved in 20 mL of 0.1 M NaHCO_3_) were added into the residue in the tubes. After thorough mixing, the solutions were dialyzed in 150 mL 0.1 M NaHCO_3_. The samples were incubated in a shaking water bath at 37 °C for 3 h. The contents of the dialysis bags were transferred to sterile centrifuge tubes, than heated in a boiling water bath for 5 min in order to inactivate digestive enzymes. After cooling to 20 °C, the solutions were centrifuged (3024× *g*, 10 min, 4 °C). Finally, the supernatants were decanted and lyophilized (large intestine phase). The sodium bicarbonate sub-dialysis solution was also lyophilized (small intestine phase). The digestions were performed in triplicate. A control sample, which consisted of the gastrointestinal juices, enzymes, and water instead of sample extract, was also taken into account in the experimental analyses to evaluate the possible influence of the digestive enzymes on the results.

### 2.5. Cell Cultures 

Both, mouse embryonic fibroblasts (NIH-3T3) and mouse macrophage-like RAW 264.7 cells were purchased from American Type Culture Collection (ATCC, Manassas, VA, USA). Cells were maintained in DMEM (Dulbecco’s modified Eagle’s medium) and supplemented with 10% fetal bovine serum (FBS) (Gibco™, Grand Island, NY, USA) and 1% penicillin-streptomycin. Human microvascular endothelial cells (HMEC-1), a gift from Kathryn Kellar, Centers for Disease Control and Prevention, Atlanta, GA, USA), were cultured in MCDB 131 medium with fetal bovine serum (10% *v*/*v*), glutamine (2 mmol L^−1^), epidermal growth factor (10 ng mL^−1^), hydrocortisone (1 μg mL^−1^), and penicillin-streptomycin. All cell culture experiments were performed in a humidified 5% CO_2_ and 95% atmosphere at 37 °C. Tissue culture plastics were supplied by Greiner Bio-One GmbH (Frickenhausen, Austria). All the experimental measurements were performed using the Synergy 2 Microplate Reader (BioTek Instruments Inc., Winooski, VT, USA).

### 2.6. Cell Line-Based Studies

#### 2.6.1. Cell Metabolic Activity

Cell cytotoxicity was assessed using a 3-[4,5-dimethylthiazol-2-yl]-2,5-diphenyltetrazolium bromide (MTT) assay [32], NIH-3T3 and HMEC-1 cells were seeded in 96-well plates at density of 104 cells/well and treated with various concentrations (0.15–5.00 mg mL^−1^) of microcapsules. After removing the media, MTT (5 mg mL^−1^) was added and incubated at 37 °C for 3 h. Supernatants were carefully collected and 50 μL of dimethyl sulfoxide (DMSO) was added. After incubating at 25 °C for 30 min, the reactants were measured by a microplate reader (UV max, Molecular Devices, TN, USA) at 570 nm. 

In case of RAW 264.7 cells, the effect of samples on cell viability was assayed with the PrestoBlue™ reagent. The cells were seeded into a 96-well plate at a density of 104 cells/well overnight. Next day after reaching confluence, cells were treated with samples at the highest noncytotoxic concentration IC_0_ equal to 0.15 mg mL^−1^ for 24 h. Then, the PrestoBlue reagent was added for 30 min and fluorescent signal at F530/590 nm was measured. Cell metabolic activity (cytotoxicity) was calculated as percentage of value obtained for cells incubated with samples in comparison to control cells treated with medium.

#### 2.6.2. Cell Migration Assay 

NIH-3T3 and HMEC-1 cells were seeded in 12-well plates at a density of 2 × 10^5^ cells well^−1^ in 10% serum containing DMEM/MCDB 131 medium for 6 h. Then, the medium was changed to FBS-free and incubated with various concentrations (0.15–5.00 mg mL^−1^) of microcapsules for the next 24 h. To eliminate cell proliferation, the cells were pretreated with 10 µg mL^−1^ mitomycin C (Sigma, St. Louis, MO, USA) for 1 h and washed with phosphate-buffered saline (PBS) prior to scratching. The scratching was performed by scraping with a sterile rubber policeman. The wound margins were photographed at time t = 0 under a phase-contrast microscope (400 × magnification) and imaged using a digital camera (Olympus America Inc., San Jose, CA, USA). Cells were treated with samples for up to 24 h with cell media only (control) or with 0.15 µg mL^−1^ of microcapsules, HH or RJ dissolved in culture medium. The concentration range of RJ (0.15 µg mL^−1^) was based on RJ concentration with >90% cell viability by MTT assay in a preliminary experiment. The scratched area was subsequently re-photographed in the same field at different time points (3, 6, 12 and 24 h). The experiments were performed in triplicate for each of four independent trials. Cell migration was analyzed by ImageJ (image processing and analysis in Java by NIH image) and expressed as percent of wound/scratch area coverage by cells moving into the scratched wound area at different time points.

#### 2.6.3. Detection of Intracellular Reactive Oxygen Species Generation in RAW 264.7 Cells 

The RAW 264.7 cells were seeded into a 96-well plate at a density of 10^4^ cells/well. After the cells’ treatment with samples, the cells were washed with PBS and incubated with DMEM and 10 µM dichlorodihydrofluorescein diacetate (DCFH-DA) dye. Fluorescence intensity at F485/530 nm was determined after 30 min incubation. 

#### 2.6.4. Determination of TNF-α, IL-6, and Nitric Oxide Secretion

To perform the experiment RAW 264.7 cells were seeded into a 24-well plate at a density of 5 × 10^4^ cells well^−1^. To test the effect of the preparations in the case of chronic inflammation, cells were treated with the preparations for 2 h, and then stimulated with 1 µg mL^−1^ of lipopolysaccharide (LPS) O55: B5 from Escherichia coli for 18 h. Untreated cells and LPS were the negative control of inflammation, and cells treated with LPS without samples were the reference model of inflammation (positive control). After the cells’ treatment with samples for 24 h, the medium was collected and centrifuged at 150× *g* for 5 min to precipitate the cell debris. In the supernatants the protein concentrations of IL-6 (Mouse IL6 ELISA kit, Biorbyt Ltd., Cambridge, UK) and TNF-α (Mouse TNFalpha ELISA kit, Biorbyt Ltd., Cambridge, UK) were determined using ELISA kits, following the manufacturer’s instructions. The accumulation of NO metabolite in the cell culture supernatant was measured using Griess reagent (1% sulfanilamide and 0.1% naphthylethylenediamine dihydrochloride; Sigma Aldrich), where an equal volume of the supernatant was mixed with Griess reagent in a 96-well plate. After incubation at room temperature and darkness for 15 min, the absorbance was measured at 540 nm. 

### 2.7. Statistical Analysis

Unless stated otherwise, all the biological results are presented as means of 3–12 repeated experiments ± SD. All calculations were evaluated for significance using one or two-way ANOVA followed by Dunnett’s test with the GraphPad Prism 6.0 software (GraphPad Software Inc., La Jolla, CA, USA). *p* ≤ 0.05 was considered statistically significant.

## 3. Results and Discussion

### 3.1. Antioxidant Activity

#### 3.1.1. Antioxidant Activity by Electron Paramagnetic Resonance (EPR) Spectroscopy

**Figure 1:**
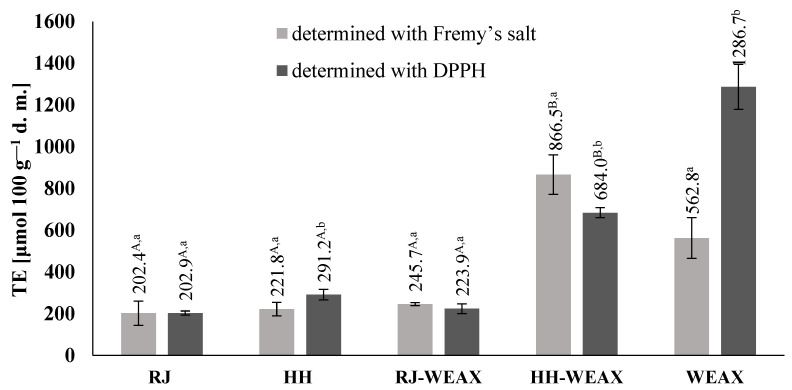
Antioxidant activity of encapsulated bee products analyzed by EPR spectroscopy. RJ-royal jelly; HH-honeydew honey; WEAX-water extractable arabinoxylans; TE-trolox equivalens. Uppercase letters followed by different letters indicate significant differences in antioxidant activity in the microcapsules obtained after spray drying bee products with WEAX depending on the type of the core material used for their preparation (*n* = 3; *p* ≤ 0.05); lowercase letters followed by different letters indicate significant differences in antioxidant activity in the individual sample depending on the type of radical used in the assay (*n* = 3; *p* ≤ 0.05); data are presented as mean ± SD.

The native honeydew honey, royal jelly, and its microcapsules were subjected to EPR analyses in order to measure their radical-scavenging ability against stabilized Frémy’s salt and DPPH. Semi-stable radicals of Frémy’s salt and DPPH can react with antioxidants of bee’s products along different pathways; the predominant reaction represents hydrogen/electron transfer from the antioxidant [33,34]. The signal intensity is proportional to the number of spins and, thus, can be used to measure the relative concentration of the paramagnetic species. As different assay conditions result in scavenging of different radical species, the values obtained for antioxidant activity are not directly comparable, but they are expected to show the same tendency. The results of the antioxidant activity measured as ability to scavenge Frémy’s salt and DPPH (calculated via calibration curve from the content of Trolox) for native honeydew honey, royal jelly, rye bran WEAX, honeydew honey-loaded, and royal jelly loaded WEAX-based microcapsules indicate a significant increase in antioxidant activity as a result of microcapsulation process (Figure 1). The results obtained for the microcapsules are in the range 245.7–866.5 μmol TE 100 g^−1^ d.m. for the Frémy’s salt-assay and 223.9–683.9 μmol TE 100 g^−1^ d.m. for the DPPH-assay. A proportional correlation (R^2^Fremy’s; DPPH = 0.99) in antioxidant activity was observed for all samples. The obtained bee product microcapsules indicated comparable or even higher ability to scavenge the stabilized radical of both Frémy’s salt and DPPH, than native bee products. The antioxidant activity of HH-WEAX was 290.7% and 134.9% higher for Frémy’s salt and DPPH assays, respectively, than the antioxidant activity of native honeydew honey. There was no statistically significant difference in the antioxidant activity determined for the RJ-WEAX capsules and native royal jelly, both for Frémy’s and DPPH assay. Due to the high antioxidant activity of the WEAX carrier material itself (562.8 μmol TE 100 g^−1^ d.m. against Frémy’s salt radicals and 1286.7 μmol TE 100 g^−1^ d.m. against DPPH radical), its use has a positive effect on the antioxidant activity of end products. It is worth noting that the amount of carrier in each type of microcapsules obtained is only 23.8% and 11.1% for the HH-WEAX and RJ-WEAX preparations, respectively. The increase in the antioxidant activity in the microcapsules must be the result of the synergistic effect of the bee products and the carrier fraction. The use of rye bran WEAX as carrier seems to favor the transformations that led to better electron-transfer ability of the bioactive compound found in the core material. 

At the same time, a significant dependence of the antioxidant activity of the obtained fixed preparations of bee products on the type of encapsulated core material was found. Compared to native bee products, the highest scavenging capacity of both Fremy′s salt radicals and DPPH was determined for preparations whose core material was honeydew honey (HH-WEAX). The comparison of antioxidant activity by EPR spectroscopy shows that the encapsulation process allows not only to maintain, but even to increase the original antioxidant properties of bee products in core material of microcapsules. From this result, it is possible to conclude that the application of the rye bran WEAX as a carrier exhibits protective effect of bee products antioxidants during encapsulation process and due to high antioxidant activity of carriers has a positive impact on antioxidant activity shown for both HH-WEAX and RJ-WEAX. 

As reported by Zalibera et al., the antioxidant activity for honeys of various floral origins ranges from 15–114 μmol TE 100 g^−1^ [35]. According to Gheldof and Engeseth, the ORAC value of honey is strongly dependent on its floral source and takes values in the range 320–1630 μmol Trolox equivalent 100 g^−1^ honey [36]. The native honey Frémy’s salt (221.8 μmol TE 100 g^−1^ d.m.) and DPPH (291.2 μmol TE 100 g^−1^ d.m.) antioxidant activity was in a range characteristic for honeys, thus consistent with the literature data. Samborska et al. determined the antioxidant activity of honey powders obtained after dehumidified air spray drying with maltodextrin, nutriose and mixture of both carriers [37]. The values range from 129.4 and 139.5 to 144.4 μmol TE 100 g^−1^ d.m., respectively. Tomczyk et al. obtained honey powders using maltodextrin as carrier (dry mater mass ratio 1:1) [38]. Honey powders were characterized by high antioxidant activity ranging 59.05–182.67 μmol TE 100 g^−1^ d.m. 

Čeksteryté et al. studied the antioxidant potential of pure bee product, where the values of DPPH and TEAC scavenging capacity of royal jelly were equal to 327.6 and 615.3 μmol TE 100 g^−1^ d.m., respectively [39]. According to the study of Ecem Bayram et al., Turkish royal jelly samples exhibit high antioxidant activity of 1522.2–2081.6 μmol TE 100 g^−1^, and thus, the values of native royal jelly analyzed in this study (Frémy’s salt 202.38 μmol TE 100 g^−1^ d.m. and DPPH 202.90 μmol TE 100 g^−1^ d.m.) are lower than values presented in the literature [40]. 

There are no data on the antioxidant activity of fixed royal jelly preparations in the literature. It is commonly claimed that thermal treatment of bee products such as honey or royal jelly leads to chemical degradation of their bioactive ingredients [36,41,42,43]. However, an increase in the antioxidant activity of bee products because of heat treatment may also be observed. The evaluation of the short-term thermal treatment of honey showed no statistically significant changes for bioactive properties [44]. Regarding the qualitative and quantitative analysis of phenolic compounds, short-term thermal treatment such as spray drying may lead to some modifications, resulting in new compounds creation, thus positively impacting the antioxidant activity. Additionally, this type of processes may lead to release of bound phenolic acids such as gallic acid, which content appears to be higher in thermally treated samples than in native ones, resulting in enhancing of radical scavenging potential [45]. This phenomenon may be explained by the presence of thermally degradable hydrolysable complex phenolic compounds in native bee’s products—these chemicals may be degraded under high temperature, leading to the increase in free phenolic compounds and, consequently, the intensification of the antioxidant activity of thermally treated samples, in this case microcapsules [46]. Moreover, during thermal treatment, FA, which is derived mainly from WEAX, can be degraded to guaiacol, 4-methylguaiacol, 4-ethylguaiacol, and 4-vinylguaiacol [47]. It is known that 4-vinylguaiacol can participate in the so-called Maillard reaction, forming other bioactive adducts that enhance the antioxidant activity of microcapsules. As presented earlier, the process of honey microencapsulation may work in favor of antioxidant properties of microcapsules [24]. As also shown, the encapsulation process enhanced the DPPH radical scavenging activity of the microcapsulated honey by 52%.

#### 3.1.2. Cu^2+^-Ion Chelating Capacity of Microcapsules

**Figure 2:**
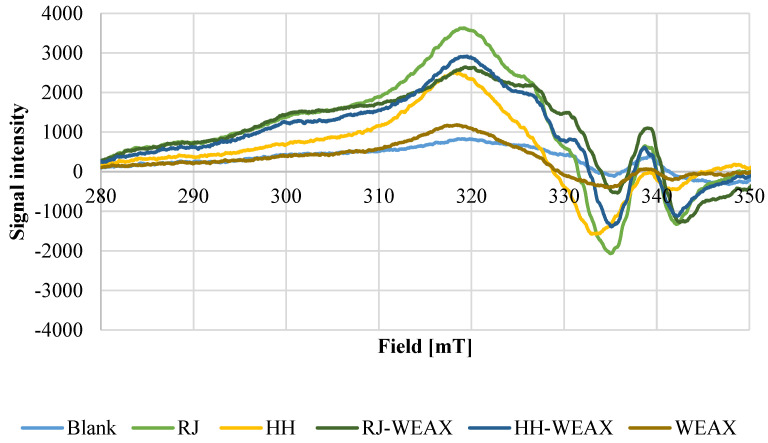
Cu^2+^-Ion chelating capacity, pH 7.4, incubation time 1 h.

Antioxidant properties of bee products can result also from their ability to chelate transition metal ions, such as Cu^2+^. Metal ions can generate reactive oxygen species (ROS) by Fenton or Haber–Weiss reaction. In the Fenton reaction the hydroxyl radical (HO^•^) is produced from hydrogen peroxide. In the iron-catalyzed Haber–Weiss reaction the superoxide radical (O^2•−^) reduces ferric to ferrous ions, which then are again involved in generating of hydroxyl radicals [48]. High reactive hydroxyl radicals can interact with many biologically important cellular components and, therefore, lead to lipid peroxidation, DNA damage and polymerization, or denaturation of proteins [49]. The chelating of transition metal ions such as Cu^2+^ to phenolic compound of bee products can stabilize prooxidative activity of those ions. Copper (II) ions provide a satisfactory signal-to-noise ratio in EPR and in contrast to iron (III) does not cause difficulties in EPR measurement due to ubiquitous distribution of iron. However, the pro-oxidant behavior of both metal ions as well as its inhibition by chelating agents is based on the same mechanism: The chelated ions contribute less to radical generation by Fenton or Haber–Weiss reaction and the trends observed by this method for Cu^2+^ can be expected as well for Fe^3+^ [50]. As trace metals are mainly absorbed in the small intestine, within which the pH level is about 6.6–7.5, the chelating activity against Cu^2+^ was examined at a comparable value of pH 7.4, after 1 h of incubation time by means of EPR. The corresponding spectra at pH 7.4 are shown in Figure 2. In the slightly basic pH levels, Cu^2+^ ions precipitate as hydroxide, and no signal can be seen in the spectrum of the blank value. Strong complexing agents such as RJ, HH, HH-WEAX, or RJ-WEAX are able to keep copper (II) ions in solution even at this pH value, leading to the complex signal generation in the EPR spectrum. In the EPR spectra of the complexes, the maximum of the Cu^2+^ signal is retained for all samples except rye bran WEAX. However, the amplitude is slightly increased for native royal jelly, HH-WEAX and RJ-WEAX microcapsules. The increase in amplitude indicates increased copper complexation. In addition, in all samples (except WEAX) new signals are generated in the area of the minimum, what may indicate an increased complexation of copper.

The presented spectra indicate Cu^2+^ chelating activity of bee products and their microcapsules. However, this is the first report demonstrating the metal complexing activity of honeydew honey, royal jelly, and encapsulated bee products. According to Aazza et al., honeys are characterized by metal chelating activity expressed as IC_50_ value ranging from 13.29–91.81 mg mL^−1^ [51]. The capacity to complex trace metal ions does not correlate with total phenol content.

### 3.2. Simulated Gastrointestinal Digestion

In order to analyze the potential application of microcapsules containing honeydew honey or royal jelly as core material in controlled release/delivery systems, an in vitro gastrointestinal digestion was carried out. The quantity and profile of phenolic compounds present in WEAX and HH, RJ and their microcapsules (RJ-WEAX and HH-WEAX) after in vitro gastrointestinal digestion process were determined and compared (Table 1). In regard to the digested fractions, both the gastric and small intestine fractions, which should represent the bio-accessible fraction capable of reaching the blood flow, and the large intestine fraction, which is metabolized inside the colon by the microbiota or eliminated through the feces, were analyzed. Bioactive substances found in royal jelly and honey represent two groups of phenolic compounds such as phenolic acids and flavonoids. The most prominent representatives of these compounds are derivatives of cinnamic and benzoic acids such as sinapic acid, gallic acid, ellagic acid, and 3,4-dihydroxybenzoic acid, and flavanols such as (+)-catechin and (−)-epicatechin. These phenolic compounds were also some of the major phenolic compounds found in different honeydew honeys’ origins such as Tilia platyphyllos, Quercus robur, [52] and Abies alba [53].

The in vitro digestion analysis showed that the release rate of total phenolic compounds in the gastric phase was 61.4% and 50.5% higher for native honey and royal jelly, respectively, than for their microcapsules. Following the gastric digestion, release rates of elagic acid, (+)-catechin and (−)-epicatechin were significantly (*p* < 0.05) higher for native honeydew honey and royal jelly than HH-WEAX and RJ-WEAX microcapsules. 

The small intestine fraction of digested microcapsules of bee products contained on average 49.50 mg of flavonoids, 36.80 mg of hydroxybenzoic acids, and 78.87 mg of hydroxycinnamic acids 100 g^−1^ d.m., while in the small intestine fraction of native bee product was determined on average 0.20 mg of flavonoids, 7.60 mg of hydroxybenzoic acids, and 6.10 mg of hydroxycinnamic acids 100 g^−1^ d.m.

Moreover, the release rates of sinapic acid, (−)-epicatechin and 3,4-dihydroxybenzoic acid in small intestine were 14, 14, and 377 ‘620 and 9, respectively; this was 49-fold higher for microencapsulated honey and royal jelly, respectively, than in their native forms. The release rate of total phenolic compounds in small intestine was increased by the microencapsulation process by 877% and 1632% for HH-WEAX and RJ-WEAX, respectively, suggesting that the bee products microencapsulation provided intestine-specific delivery system. Furthermore, the use of rye bran WEAX carrier resulted in a higher release of caffeic acid, chlorogenic acid, *p*-coumaric acid, FA, sinapic acid, gallic acid, 3,4-dihydroxybenzoic acid, 3-hydroxybenzoic acid, elagic acid, procyanidin B, and quercetin-3-galactoside in the large intestine, and then amounts of phenolic compounds were released as a result of digestion of native bee products.

The phenolic compounds occur as unbound monomers, i.e., aglycones, or in bound form as esters or glycosides. Monomers of phenolic compounds are more labile under the conditions of the digestion process than their covalent or non-covalent complexes with carbohydrates or proteins. Therefore, monomers are more easily degraded due to pH changing and due to the action of enzymes. The antioxidant activity of the flavonoid monomers and phenolic acids present in native honey or royal jelly depends on the pH value and is inversely proportional to its increase. Moreover, the efficiency of the phenolic glycoside hydrolysis process highly depends on the type of saccharide constituting the O-glycoside molecule. Unlike polysaccharide conjugates, glucose or fructose conjugates dominating in native bee products are rapidly hydrolyzed by the action of gastric acid and may be easily degraded.

Due to the type of heteropolysaccharide biopolymers used in microencapsulation process, the phenolic compounds found in the core material may interact with polysaccharide compounds to form a covalently bonded network that does not undergo rapid hydrolysis. Covalent phenol-polysaccharide complexes may also be characterized by poor solubility or even lead to an increase in the viscosity of the digestive fluids, which in turn may reduce the diffusion of bioactive compounds in the gastric and small intestine phase. Less complex conjugates can be transported directly by previously undefined carbohydrate-phenol transporters located in the brush border of the small intestine. On the other hand, high-molecular complexes of phenolic compounds can reach the large intestine, where they will be metabolized by the microbiome to monomers, often with higher antioxidant activity than the original high-molecular complexes.

Therefore, despite the decrease in some phenolic compounds after gastric digestion, the phenolic compounds of HH-WEAX and RJ-WEAX microcapsules still showed higher stability during the simulated digestion, including the duodenal phase.

In the next step, a significant influence of the type of the core material of microcapsules on the biostability of the released phenolic compounds in relation to the conditions prevailing at individual digestion stages was observed. The process of honey encapsulation contributed to the most effective reduction in the amount of phenolic compounds released in the stomach and increased bioavailability of bioactive compounds in the large intestine by 61 and 4997%, respectively, compared to the amount of compounds released as a result of native honey digestion. On the other hand, microcapsulation of royal jelly resulted in the highest increase in the bioavailability of phenolic compounds in the small intestine fraction, increasing to 1642% in relation to the amount of compounds released after RJ digestion. 

Cianciosi et al. obtained comparable results of total amount of phenolic compounds in the eliminated (large intestine) fraction of digested manuka honey (0.52 mg GAE g^−1^ honey); however, much lower amounts in the bio-accessible (small intestine) fraction (0.20 mg GAE g^−1^ honey) [54]. Similar results were obtained in studies by Seraglio et al., who evaluated the total content of phenolic compounds before and after honey digestion in different types of Mimosa scabrella Bentham honeydew honeys, where the total content of phenolic compounds in both the gastric and the duodenal fraction was 115.9–144.4 mg GAE 100 g^−1^ and 58.3–81.3 mg GAE 100 g^−1^, respectively [53]. There is no data in the literature on the release rate of phenolic compounds of digested honey or royal jelly microcapsules. Suhag et al. determined total phenolic content of spray dried honey powder containing different carriers [55]. The honey powder dried with maltodextrin was characterized a total phenolic content of by 42.72 mg GAE 100 g^−1^. Honey powder dried with maltodextrin and fortified with aonla and basil extract showed an increased amount of total phenolic compound by 37.2%. On the other hand, He et al., using arabic gum and gelatin as a carrier, encapsulated royal jelly sieve residue, which consisted mainly fatty acids of royal jelly’s lipid composition [56]. The obtained microcapsules were subjected to in vitro digestion in order to investigate the final release rate in the intestinal phase. The experiment showed a 32.9% release rate of phenolic compounds in the stomach and 65.8% release rate in the small intestine environment, indicating that the microcapsules have gastric environmental tolerance and enteric solubility. In the study of Keskin et al., ethanol extract of propolis was encapsulated using sodium-alginate as a coating material. The encapsulation process of ethanol propolis extract contributed to controlled release of propolis active components, as it allowed us to significantly increase the release rate of the beads in simulated gastric and intestinal systems [57]. In another study Kesikin et al., aimed to encapsulate propolis active constituents on the surface of whole pollen grains. The authors achieved good immobilization efficiency of 53%. Moreover, obtained pollen–propolis beads contained more phenolic compounds and were characterized by higher ferric reducing activity than native pollen and propolis. The study of in vitro simulated digestive systems showed higher release rate of bioactive compounds as a result of immobilization process of pollen–propolis compared to native bee products [58].

It has been demonstrated that the food matrix plays a significant role in the biostability of polyphenols during the digestion process. The ability of phenolic compounds to bind to other dietary components, such as polysaccharides and proteins [59], may protect them from their possible chemical degradation [60]. Besides that, high stability of some compounds in the gastric and intestinal conditions reported for pure compounds, such as (+)-catechin, quercetin, FA, and rutin [61], also supports potential sustaining of the biological activity of samples after their digestion. 

In summary, analyses of the profile of phenolic compounds released at individual stages of the digestion process demonstrate that phenolic compounds of bee products as core materials in capsules showed higher stability during simulated digestion at the gastric stage, which led to their higher bioavailability in the section the small intestine, where bioactive compounds are absorbed into the bloodstream. It should also be emphasized that the bioactive compounds present in the rye bran heteropolysaccharides carrier showed significant resistance to the adverse effects of the conditions in the stomach. On this basis, it can be concluded that the applied conditions of the encapsulation process and the type of carrier allowed the creation of systems for the controlled delivery of bioactive compounds to the selected matrix—the small intestine. This solution may have potential future use in the development of systems for controlled and targeted drug delivery.

### 3.3. The Samples Encapsulation Influence on the Metabolic Activity and Migration of NIH-3T3 and HMEC-1 Cells

**Figure 3:**
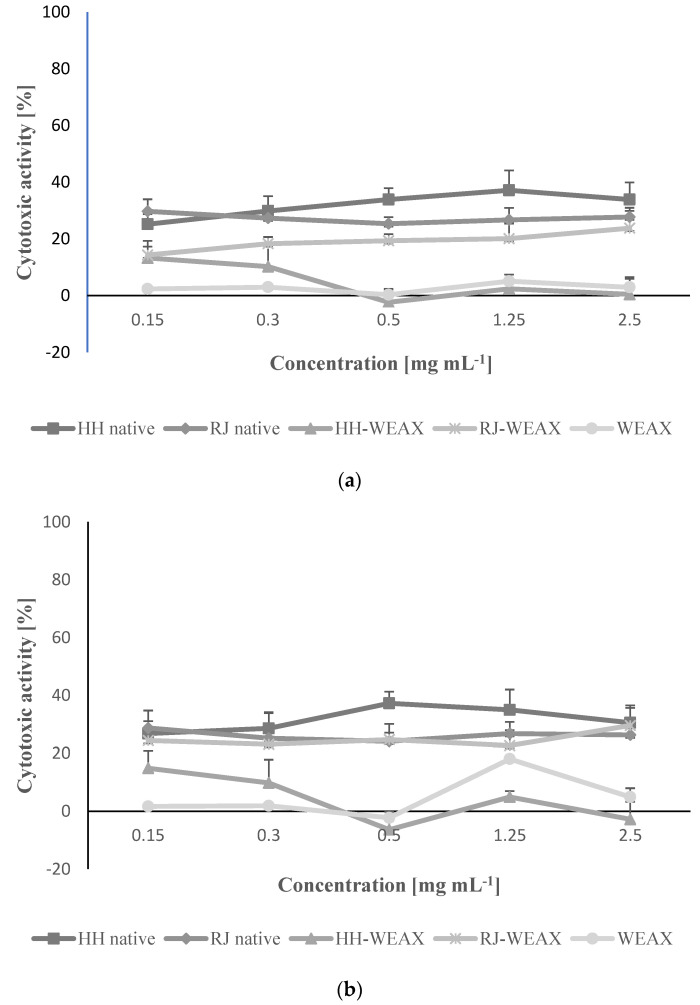
Assessment of cytotoxic activity of bee products encapsulated with WEAX biopolymer at 0.15 mg mL^—1^ concentration against NIH-3T3 (**a**) and HMEC-1 (**b**) cells, determined with the MTT assay. Each value on the graphs represents the mean value ± SEM, *n* = 3.

First, the impact of samples using concentrations of 0.15–2.50 mg mL^−1^ on metabolic activity of NIH-3T3 and HMEC-1 cells was studied with MTT assay. As presented in Figure 3, the range of cytotoxic activity differed between the analyzed samples. The lowest influence on metabolic activity in both studied cell lines had WEAX and HH-WEAX, which even at the highest concentration decreased metabolic activity by 5–10%. Other samples, RJ-WEAX, HH and JR, had a stronger effect, inducing cytotoxic effect by 20–30% at the concentration 2.50 mg mL^−1^. Still, an increase in cytotoxicity was not followed be the increase in concentration. Perhaps this was due to the action of the hydrogen peroxide produced in honey by the enzyme glucose oxidase when in contact with tissue [62]. Hydrogen peroxide is most likely responsible for the antibacterial effect of honey, but it can also, depending on the concentration, have a slightly cytotoxic effect on eukaryotic cells. This effect may have been influenced by the low pH of native preparations, which increased after encapsulation process with arabinoxylans. Based on these results the samples influence on cell migration was determined for the highest non-cytotoxic concentration equal to 0.15 mg mL^−1^.

**Figure 4:**
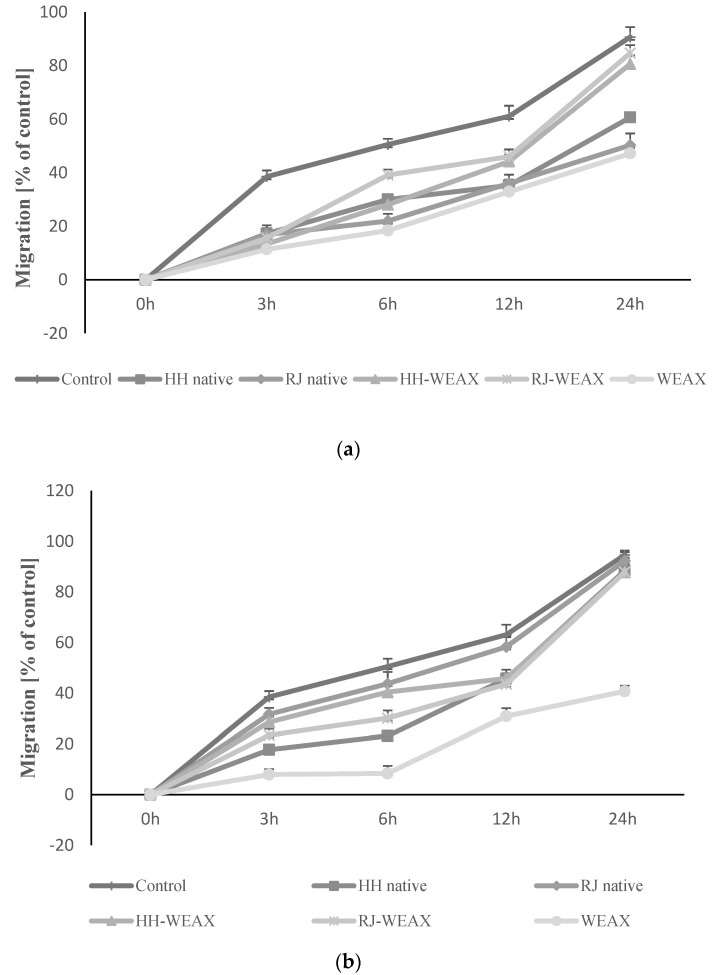
Assessment of cytotoxic activity of bee products encapsulated with WEAX biopolymer at 0.15 mg mL^—1^ concentration against NIH 3T3 (**a**) and HMEC-1 (**b**) cells. Each value on the graphs represents the mean value ± SEM, *n* = 3.

Wound healing is an essential physiological process important for tissue homeostasis, but it can be disturbed in the course of the disease and contribute to many pathologies. The skin is a complex tissue, so a “full-thickness” wound causes damage to many structures and individual cells, including (from outside to inside) the following: the epidermal keratinocyte layer (the body’s barrier against the outside world) with accompanying appendages; hair follicles and sweat glands; basement membrane; the dermis, which is a complex structure consisting of fibroblasts, the extracellular matrix, nerves, and blood and lymph vessels [61]. Both honey and royal jelly are commonly used to stimulate the wound healing process, also in a clinical setting; however, the relationship between its healing properties and the wound healing mechanism is still largely unexplored [63]. On this basis, the experiment characterizing the impact of the encapsulation process on the rate of wound healing took into account two types of cells, HMEC-1 and NIH-3T3, which play a major role in the reconstruction of blood vessels and skin, respectively. The effect of samples on the migration rate of human endothelial cells and mouse fibroblasts was determined by scratch migration assay, which simulates the wound healing process in vitro (Figure 4a,b). Analysis of the rate of migration of NIH-3T3 cells showed a significant effect of time on the % of wound closing (Figure 4a). The rate of cell migration increased in proportion to the incubation time. It was observed that fibroblasts treated with encapsulated bee products were characterized by a higher rate of cell migration (on average by 20% and 30%) compared to the migration of cells incubated with native honey and royal jelly. The analysis also showed a comparable to the control cells (90%) migration rate of NIH-3T3 cells treated with RJ-WEAX (85%) and HH-WEAX (80%) in a time-dependent manner. For comparison, fibroblasts treated with native honey and royal jelly at 24 h of the experiment achieved only 60% and 50% of migration rate, respectively. Strong reduction in the migration rate of the NIH-3T3 cells in comparison to the control was found for the biopolymers WEAX (47%). There was no significant difference in the impact of the type of core material (HH or RJ) of the analyzed encapsulates on the cell migration rate. The highest increase in the encapsulated migration rate was observed within the 12–24 h period of the experiment.

**Figure 5:**
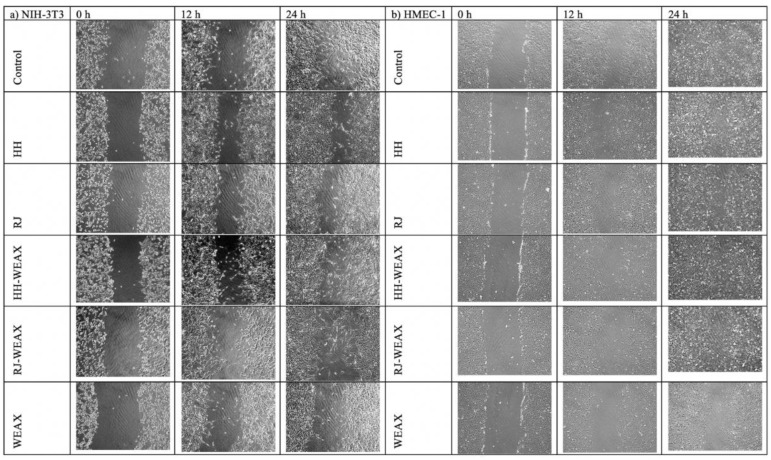
Assessment of the migration rate of (**a**) NIH-3T3 and (**b**) HMEC-1 cells for the HH-WEAX microcapsules at 0.15 mg mL^−1^ concentration in comparison to the control sample; 400× magnification.

The analysis of the results showed a comparable to the control sample (95%) rate of migration of HMEC-1 cells treated with native RJ (92%), HH-WEAX (87%) and RJ-WEAX (88%) in a time-dependent manner (Figure 4b). The microphotographs showing the changes in the cells migration of NIH-3T3 and HMEC-1 cells after 12 and 24 h incubation with microcapsules are presented in Figure 5a,b.

More than two- and three-fold reductions in the rate of cell migration in the HMEC-1 cells compared to the control sample was demonstrated for the WEAX biopolymers (41%–24 h). WEAX heteropolysaccharides in an aqueous medium lead to gelation of the solution due to the crosslinking reaction. The decreased migration rate of HMEC-1 cells due to the action of WEAX biopolymer may be caused by an increase in the viscosity of the medium in which the cells migrate. The migration rate of HMEC-1 cells treated with the HH-WEAX preparation in the first 3 and 6 h of the experiment was 10% and 20%, respectively, compared to cells treated with HH native. The rate of cell migration increased significantly after 12 and 24 h of analysis, finally reaching 87% (24 h).

After 24 h, cells treated with microcapsules migrated at the same rate as the control cells, which may indicate the lack of differentiation of both the endothelium and fibroblasts into myofibroblasts that are characterized by greater migration potential, and thus both sample and control cells indicate lower potential for scarring and tissue fibrosis in the process of wound healing and overgrowth [64,65]. A study by Majtan et al. showed that selected honey flavonoids from fir honeydew inhibit TNF-α-induced MMP-9 expression in human keratinocytes, reducing wound inflammation [66]. Clinically, there have been numerous observations that honey reduces swelling and exudation, minimizes scarring, and has a soothing effect when applied to inflammatory wounds and burns, and not only increases the rate of wound growth [65].

A lower stimulation of the migration process was determined by Tan et al., who analyzed the effect of Tualang honey concentration on the migration rate of HCEP cells (Human Corneal Epithelial Progenitor Cell Line) [67]. The migration rate of HCEP cells after 48 h of incubation increased depending on the concentration of honey. The highest migration, amounting to 17.3% and 20.5%, was recorded for HCEP cells treated with 0.04 and 0.4% Tualang honey, respectively, compared to 12.6% of migration for the control sample. Ranzato et al. obtained a significant stimulation of the migration of HaCaT cells (immortalized human keratinocyte line) under the influence of buckwheat, manuka and acacia honey [68]. Cells treated with each of the three types of honey showed more than double the wound healing rate after 24 h of incubation compared to control. Kim et al. analyzed the effect of royal jelly on the migration of human dermal fibroblasts (HDFs) [69]. Royal jelly in concentrations of 0.1, 1.0 and 5.0 mg mL^−1^ showed the most favorable effect on cell migration after 20 h of the experiment. It is worth noting that in the 8 h of the experiment, the concentration of 5.0 mg mL^−1^ royal jelly showed over 10% higher cell migration rate than the control sample, which was not demonstrated for the concentrations of 0.1 and 1.0 mg mL^−1^. In the 20th hour of the analysis, migration of HDF cells it reached 60, 82, and 80% for concentrations of 0.1, 1.0, and 5.0 mg mL^−1^, respectively. The studies presented by Prasathkumar et al. discussed the effect of manuka honey enclosed in a chitosan dressing on the migration rate of wound cells in an animal model [70]. Wounds treated with the manuka honey dressing showed 100% cell migration in 18 d of the experiment as opposed to the control which achieved complete wound closure in 21 d of the study.

As presented above, the microcapsules of bee products may have a beneficial effect on the migration rate of endothelial cells. High concentrations of glucose and fructose, the main components of honey and royal jelly, are the optimal source of energy for cells, supporting the rate of migration. The process of cell migration is highly dependent on metabolism and epithelial glycogen stores as the main source of energy [71]. It has been documented that an injury results in stimulation of the expression of glucose transport protein 1 (GLUT1) in cell membranes, which aims to increase glucose transport into cells [72]. Simple sugars available in bee products and their encapsulates can therefore provide additional energy resources through GLUT1, accelerating the migration of HMEC-1. This phenomenon explains the significant slowdown in the rate of migration reduction observed for the WEAX biopolymers, which mainly contain polysaccharide chains.

In addition, honey and royal jelly exhibit antimicrobial properties. A bacterial infection adversely affects the wound healing process. The most important antibacterial factors of honey include the following: low water activity and low pH, high sugar content resulting in high osmotic pressure and the presence of enzymes, including glucose oxidase, which in the presence of water transforms glucose into gluconic acid and H_2_O_2_ [73,74]. H_2_O_2_ degrades the components of cell membranes and proteins, and therefore has an antibacterial effect. The antibacterial properties of royal jelly are mainly due to the presence of fatty acids found in royal jelly, such as 10 hydroxydecenoic acid, 3-hydroxydodecanoic acid, 11-oxododecanoic acid and 11S hydroxydodecanoic acid. In addition, royal jelly protein, royalysin, has been identified as a bactericidal compound [75,76].

### 3.4. Immunomodulatory Properties of Microcapsules

**Figure 6:**
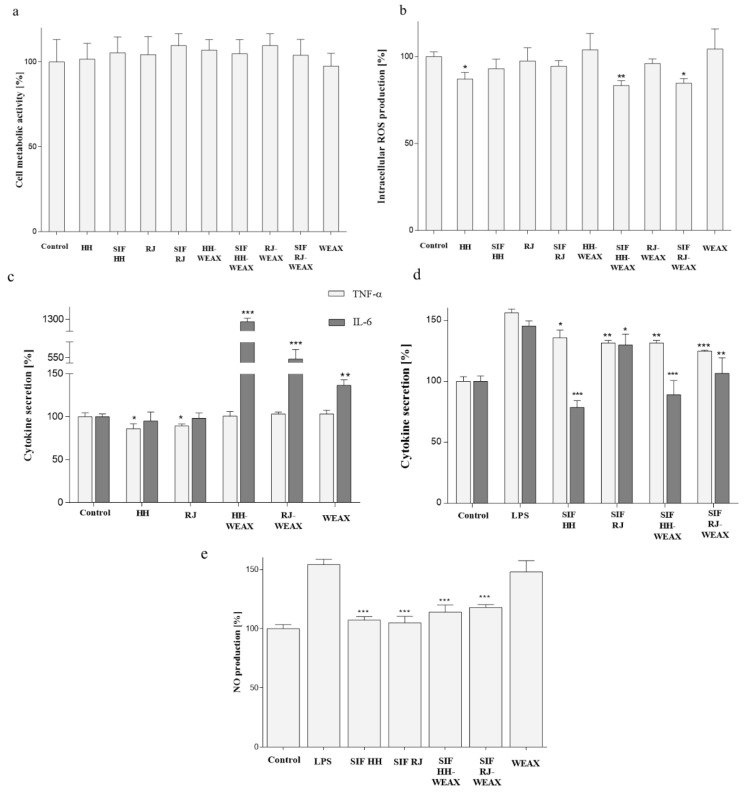
The influence of honey, royal jelly, encapsulated bee products and WEAX biopolymers at 0.15 mg mL^−1^ concentration on RAW 264.7 cells’ (**a**) metabolic activity determined with PrestoBlue assay; (**b**) intracellular ROS level determined with DCF assay; (**c**) IL-6 and TNF-α secretion determined with ELISA assay without treatment with LPS and (**d**) in cells stimulated with LPS; (**e**) NO release in LPS-stimulated cells determined with Griess reagent after 24 h incubation. Small intestine fraction (SIF) samples treated with in vitro intestinal digestion. Each value on the graphs represents the mean value ± SEM, *n* ≥ 3. Significance of differences between means * *p* < 0.05, ** *p* < 0.01, *** *p* < 0.001 versus control cells (**a**–**c**) or LPS-stimulated cells (**d**,**e**).

Inflammation is an ordered reaction of tissue to a damaging factor belonging to the infectious or chemical agents, as well as being allergies or obesity related. In inflammatory response are involved macrophages able to release many signaling substances such as cytokines [77]. Due to the positive effect of microcapsulated preparations on cells migration, next, the potential effect on the inflammatory response was studied with the murine RAW 264.7 macrophages. Cell viability was assessed using the PrestoBlue reagent, where the analysis is based on the conversion of resazurin to fluorescent resorufin in metabolically active cells. As it is presented in Figure 6a, all samples at concentration 0.15 mg mL^−1^ had no cytotoxic effect on RAW 264.7 cells. As biological effect of oral administered preparations is influenced by digestion process, analogous studies were also carried out for metabolites of the small intestine (small intestine fraction SIF) obtained in the process of simulated in vitro digestion for encapsulates obtained with WEAX biopolymers. These preparations (at corresponding concentration 0.15 mg mL^−1^) also had no negative effect on RAW 264.7 metabolic activity. 

High activity of MonoMac-6 monocytes was obtained by Tonks et al., who analyzed the immunostimulatory properties of manuka honey [78]. According to the research of Afrin et al., treatment (24 h) of human dermal fibroblast cells with strawberry tree honey and manuka honey in concentrations of 3–50 mg mL^−1^ did not significantly reduce cell viability [77]. However, it was observed that 72 h treatment with both types of honey reduces the metabolic activity of human dermal fibroblast cells in a dose-dependent manner, up to a maximum of 80%.

As elevated concentration of intracellular reactive oxygen species, also as a result of a reaction with nitric oxide, can lead to the formation of harmful peroxynitrite, which contributes to DNA and proteins damage, the effect of bee products in their native and encapsulated form on the concentration of intracellular free radicals was studied. The strongest effect on intracellular oxidative stress revealed HH-native with almost 15% decrease comparing to the control cells (Figure 6b). Among the other encapsulated samples, there was observed no significant effect on ROS regulation in RAW 264.7. Interestingly, cells treatment with digested microcapsules SIF HH-WEAX and with SIF RJ-WEAX decreased intracellular level of ROS to at least 15%. This clearly shows that both honey and royal jelly subjected to the encapsulation process with the use of WEAX carrier showed a lower effect on intracellular free radicals level than honey and royal jelly in their natural form. The antioxidant activity of honey and royal jelly seems to be the result of both the presence of phenolic compounds, and their stimulating effect on the activity of antioxidant enzymes [79,80,81,82]. It is worth commenting that antioxidant potential determined with Frémy’s salt and DPPH (Figure 1) identified microencapsulated honeydew honey (HH-WEAX) as the preparation with the highest scavenging capacity. On the other hand, in cell based study with DCF-DA probe all microencapsulated samples had comparable and low effect on intracellular ROS level. It needs to be emphasized that methods based on Frémy’s salt and DPPH use the synthetic radical cations not found in the biological system; therefore, in living cells, and from this point, the effect observed with cell lines may differ. Indeed, the cellular antioxidant capacity determined using a reduced DCF probe takes into account the bioavailability, distribution and metabolism of antioxidants within the cell and reflects the activity of the tested samples in the biological system. In our case, the honeydew honey and digested loaded microencapsulates were the most effective ROS reducers. 

The increase in the concentration of intracellular ROS, compared to the control sample, was presented by Afrin et al., who compared the effects of strawberry tree honey (STH) and manuka honey (MH) on the modulation of the concentration of intracellular reactive oxygen species in human colon cancer (HCT-116) and colon adenoma cells (LoVo) [77]. In HCT-116 cells, the highest concentration of intracellular free radicals (22% and 21%) was obtained in 48 h of the experiment for MH (20 mg mL^−1^) and STH (6 9 mg mL^−1^ and 9 mg mL^−1^), while for the control sample it was determined 6% ROS concentration. In LoVo cells, the highest level of ROS (38%) was determined for cells treated with STH at a concentration of 20 and 30 mg mL^−1^, while MH (40 mg mL^−1^) induced 34% ROS, for comparison the control sample resulted in a ROS concentration of 7%.

Among inflammatory cytokines, interleukin 6 and tumor necrosis factor α are mainly mentioned; therefore, next, the samples’ effect on their secretion was determined in RAW 264.7 cells. Analysis showed that both honeydew honey and royal jelly reduced the TNF-α level by 14% and 11%, respectively, compared to untreated cells (Figure 6c). Natural bee products did not affect the secretion of IL-6. However, it was found that the obtained encapsulates (HH-WEAX, RJ-WEAX) significantly increased the level of secreted IL-6 protein compared to native bee products. As WEAX had no effect on IL-6 secretion, observed results require more detailed future studies, for example, determination of the expression of gene encoding IL-6 on the mRNA level, or nuclear factor κB (NF-κB), which regulates the expression of proinflammatory genes, including IL-6 and TNF-α. Surprisingly, cells treated with the HH-WEAX microcapsules secreted TNF-α protein at the same level as untreated cells. The immunostimulatory effect of manuka honey was presented by Tonks et al., who observed a significant increase in the intracellular concentration of IL-6 and TNF-α of monocytes of the Mono-Mac-6 line treated with honey for 24 h [76]. The determined concentrations of IL-6 and TNF-α were 500 and 270 pg mL^−1^, respectively, while for the control sample, there was no cytokine surge [77]. Mihajlovic et al. observed the immunomodulatory effect of royal jelly (10-HDA) fatty acids against human peripheral blood mononuclear cells (PBMCs) [83]. As a result of a three-day incubation of PBMC with 10-HDA acid (50 and 500 mM), depending on the concentration, the pro-inflammatory reaction was stimulated or inhibited. The lower concentration of 10-HDA increased the level of IL-6 by 40% but did not significantly change the level of TNF-α, compared to the control. On the other hand, 500 mM 10-HDA resulted in a decrease in the concentration of both cytokines by 27% and 68% for IL-6 and TNF-α, respectively.

Taking into account the functional aspects of encapsulated bee products as food supplements, next, we checked the biological effect of all preparations after their digestion on simulated inflammation condition lipopolysaccharide (LPS)-stimulated RAW 264.7 cells. As shown in Figure 6d, in comparison to untreated cells, the LPS treatment resulted in the elevation of release of both pro-inflammatory cytokines IL-6 and TNF-α by circa 50%. All microcapsule metabolites SIF lowered IL-6 and TNF-α release in stimulated cells. The strongest effect was determined for the SIF HH-WEAX, which reduced the concentration of IL-6 by almost 50%, and decreased TNF-α by 25%. What is more, the SIF RJ-WEAX more efficiently inhibited both cytokines secretion than the SIF RF native, especially in IL-6 reduction. Therefore, it can be concluded that encapsulation of native bee products may sustain or enhance their biological activity after digestion process. In summary, the preparations subjected to the simulated digestion process showed anti-inflammatory activity against RAW 264.7 macrophages in relation to the cells stimulated with LPS. A good correlation (r^2^ = 0.74) of the ability to inhibit the IL-6 secretion with the ability to inhibit the secretion of TNF α was observed.

The relationship between the antioxidant activity and the anti-inflammatory of encapsulated bee product was observed by the correlation of the results of the analyzes of the antioxidant activity with the inhibition capacity of IL-6 and TNF-α. The scavenging capacity of the DPPH radicals (according to the EPR assay) resulted in a stronger reduction in TNF-α (r^2^_DPPH: TNF_ = –0.77). Similarly, the higher IL-6 inhibitory capacity correlated with Frémy’s salt radical scavenging capacity (r^2^_Fremy’s: IL-6_ = –0.55) and DPPH (r^2^_DPPH: IL-6_ = –0.74) as determined by EPR assay. 

During inflammation, nitric oxide is produced by inducible nitric oxide synthase (iNOS), which further enhances secretion of other proinflammatory cytokines such as TNF-α. Detection of nitric oxide (NO) production demonstrated that in cells treated with preparations after in vitro intestinal digestion, the inhibition of NO level synthesis occurred for at least 30% in comparison to cells stimulated with LPS (Figure 6e). 

Kohno et al., analyzed the anti-inflammatory potential of royal jelly by monitoring the inhibition of the release of pro-inflammatory cytokines, including IL-1, IL-6, and TNF-α as a result of stimulation of RAW 264.7 treated with LPS and interferon gamma (IFN-γ) [5]. There was a reduction in the production of cytokines as a function of royal jelly dose. The concentration of 5 mg mL^−1^ of royal jelly extract resulted in a reduction in both TNF-α and IL-6 secretion by 84.6% and 33.3%, respectively, compared to the positive control of LPS. However, for RAW 264.7 cells stimulated with LPS/IFN-γ, an average increase of 25% in IL-6 concentration was observed, relative to control. According to Ranneh et al., honey from stingless bees was found to inhibit the pro-inflammatory response of LPS-stimulated macrophages, reducing IL-6 and TNF-α levels by 44% and 23%, respectively [84]. In addition, it was proved that the honey of stingless bees showed an inhibition of interferon release up to 89% compared to the positive control. These results confirm previous in vivo analyzes, in which honey of stingless bees reduced the level of inflammatory markers, i.e., CRP, TNF-α, IL-1, IL-6, and IL-8.

Fractionation of royal jelly extract by particle size showed that the fraction responsible for the anti-inflammatory effect is composed of substances of low (<5 kDa) and high (>30 kDa) molecular weight, the former being the predominant component. Chromatographic analysis showed that MRJP3 is the compound contributing to the anti-inflammatory activity. A study by Okamoto et al. showed that the 70 kDa MRJP3 glycoprotein inhibits the production of IL-4, IL-2 and IFN-γ by T cells associated with suppression of cell proliferation [6]. Moreover, the authors proved that MRJP3 is also a molecule responsible for the inhibition of IgE and IgG1 responses in vivo. Chen et al. analyzed the anti-inflammatory effect of three main fatty acids present in royal jelly: 10-H2DA, 10-HDA and SEA [7]. The authors compared the in vitro anti-inflammatory effect of the above-mentioned fatty acids in royal jelly in macrophages of the RAW 264.7 line stimulated with LPS. The results of the studies showed that 10 H2DA, 10-HDA and SEA had a strong dose-dependent inhibitory effect on the release of the main inflammatory mediators, i.e., NO and IL-10, while only SEA decreased the concentration of TNF-α. It is also supposed to be the case that the nucleotides ATP, ADP, AMP and IMP, present in royal jelly, exhibit immunostimulatory activity [85].

The anti-inflammatory effect of honey is mainly attributed to the presence of compounds with antioxidant potential, such as phenolic compounds. In vitro studies by Kassim et al. analyzed the effect of honey extracts on the production of intracellular nitric oxide levels of RAW 264.7 mouse macrophages stimulated with endotoxins and IFN-γ [86]. It was shown that the extracts reduced the NO production in macrophages depending on the concentration of honey and the content of phenolic compounds. Similar conclusions were presented by Woo et al., who noted that chrysin inhibits the expression of COX genes in LPS-stimulated cultured macrophages, which is the result of inhibition a pro-inflammatory ejection of IL-6 [87].

## 4. Conclusions

Based on the obtained research results, it was demonstrated for the first time that the use of bioactive heteropolysaccharides isolated from rye bran as a carrier in the spray drying process of honey and royal jelly is an important factor influencing the biological and bioactive properties of fixed bee products.

Using the EPR spectroscopy for testing the antioxidant activity of the microcapsules, it was shown that the encapsulation process allowed us not only to maintain, but even significantly increase the antioxidant properties of the encapsulated bee products, compared to the activity of the native core material. Honeydew honey capsules (HH-WEAX) exhibited by 291% and 135% higher antioxidant activity for Frémy’s salt test and DPPH higher, respectively, than native honeydew honey. On the other hand, the antioxidant activity of royal jelly microcapsules did not differ significantly from the antioxidant activity of royal jelly in its native form. This may be due to the different amount of carrier added depending on the type of bee product, i.e., only 24.0% and 11.0% for the HH-WEAX and RJ-WEAX formulas, respectively. The synergistic interaction of the coating material with the core of the microcapsules was demonstrated, as well as the important role of the heteropolysaccharides used in the protection of bioactive compounds found in native bee products.

For the first time, the authors of the manuscript conducted research to determine the ability of bee products and the obtained encapsulates to chelate copper (II) ions. It was shown that the bee products encapsulates show a significantly better potential for binding copper ions compared to the carrier, i.e., bran arabinoxylans and native bee products. This fact may be reflected in the positive effect of bee product microcapsules on the microbiome.

By subjecting the innovative microcapsules of honey and royal jelly to the process of simulated digestion in vitro, on average, 85% higher biostability in the gastric stage was shown, which at the same time resulted in their higher bioavailability in the small intestine, compared to native products. Moreover, the encapsulation process allowed for the release of two to ten times more bioactive compounds in the small intestine, compared to the amount released from honey or royal jelly in their native form. The use of heteropolysaccharides from rye bran in the encapsulation of bee products enables to reduce losses both in the amount and activity of bioactive compounds of native bee products, which are mainly caused by the destructive effect of gastric acid and digestive enzymes. 

Moreover, in this study, for the first time we proved that bee products encapsulated with arabinoxylanes isolated from the rye bran can effectively ameliorate inflammatory response in LPS-treated 264.7 macrophages decreasing the secretion of IL-6, TNF-α and NO. The in vitro digestion process revealed that bee products encapsulates were stronger intracellular oxidative stress reducers, and had sustained ability to reduction in inflammation state mediators. The lack of inhibitory effect on migration rate of HMEC-1 endothelial cells and NIH-3T3 fibroblasts, the cells involved in wound healing process, identifies these preparations as agents potentially used in the management of inflammatory response.

The analysis of cell migration showed that the encapsulation of honey and royal jelly had a significantly (*p* < 0.05) positive effect on the wound healing process by stimulating the migration of both human endothelial cells of the HMEC-1 line and murine fibroblasts of the NIH-3T3 line in relation to the native bee products. The in vitro migration rate increased with the time of exposure to encapsulated preparations, which may indicate the need to use them longer than the tested 24 h. Although the migration rate of the tested cells with encapsulated preparations is not higher than that of the control cells, they can have a significant healing effect due to the gentle stimulating effect of fibroblasts, which consequently does not lead to scarring, but allows time for the action of bioactive and immunomodulating factors.

The use of bioactive heteropolysaccharide carriers in the encapsulation of honey and royal jelly enables the development of innovative systems for the controlled release of bioactive substances, which is a significant added value of the obtained microcapsules. This process also allows us to obtain bee products microcapsules with a wide spectrum of their potential applications. Potential directions of use include wound healing or nutraceuticals with immunomodulating properties, with the possibility of controlled release in the matrix.

## Figures and Tables

**Table 1:** Phenolic compounds profile during in vitro digestion.

mg 100 g^−1^ d.m.		Honey	Royal Jelly	HH-WEAX	RJ-WEAX	WEAX
**caffeic acid**	gastric	0.00 ± 0.00 ^A,a^	0.11 ± 0.01 ^A,a^	0.12 ± 0.01 ^B,a^	0.21 ± 0.01^B,a^	0.50 ± 0.02 ^a^
	small intestine	0.02 ± 0.01 ^A,b^	0.90 ± 0.03 ^A,b^	8.15 ± 0.24 ^B,b^	9.31 ± 0.28 ^B,b^	9.36 ± 0.28 ^b^
	large intestine	0.07 ± 0.03 ^A,c^	0.01 ± 0.00 ^A,c^	0.20 ± 0.02 ^B,c^	0.32 ± 0.05 ^B,c^	1.92 ± 0.06 ^c^
**chlorogenic acid**	gastric	0.03 ± 0.00 ^Aa^	0.04 ± 0.01 ^A,a^	0.52 ± 0.02 ^B,a^	0.44 ± 0.01 ^B,a^	1.25 ± 0.04 ^a^
	small intestine	0.91 ± 0.03 ^A,b^	0.87 ± 0.03 ^A,b^	1.74 ± 0.05 ^B,b^	2.45 ± 0.07 ^B,b^	2.85 ± 0.09 ^b^
	large intestine	0.01 ± 0.00 ^A,c^	0.00 ± 0.00 ^A,c^	0.56 ± 0.02 ^B,a^	0.69 ± 0.02 ^B,c^	24.85 ± 0.75 ^c^
***p*** **-coumaric**	gastric	0.09 ± 0.00 ^A,a^	0.42 ± 0.01 ^A,a^	1.49 ± 0.04 ^B,a^	0.25 ± 0.01 ^B,a^	6.16 ± 0.13 ^a^
	small intestine	0.59 ± 0.02 ^A,b^	0.73 ± 0.02 ^A,b^	3.24 ± 0.10 ^B,b^	5.14 ± 0.15 ^B,b^	14.96 ± 0.45 ^b^
	large intestine	0.09 ± 0.00 ^A,a^	0.12 ± 0.00 ^A,c^	0.92 ± 0.03 ^B,c^	0.90 ± 0.03 ^B,c^	2.52 ± 0.18 ^c^
**ferulic acid**	gastric	0.12 ± 0.00 ^A,a^	0.00 ± 0.01 ^A,a^	1.15 ± 0.03 ^B,a^	0.22 ± 0.01 ^B,a^	2.22 ± 0.07 ^a^
	small intestine	0.00 ± 0.00 ^A,b^	0.03 ± 0.01 ^A,b^	12.08 ± 0.36 ^B,b^	30.88 ± 0.93 ^B,b^	67.42 ± 2.02 ^b^
	large intestine	0.02 ± 0.00 ^A,c^	0.00 ± 0.00 ^A,a^	0.33 ± 0.01 ^B,c^	0.25 ± 0.02 ^B,a^	0.50 ± 0.02 ^c^
**sinapic acid**	gastric	0.68 ± 0.02 ^A,a^	53.71 ± 1.61 ^A,a^	1.74 ± 0.05 ^B,a^	130.80 ± 3.92 ^B,a^	1.10 ± 0.03 ^a^
	small intestine	21.66 ± 0.65 ^A,b^	34.34 ± 1.03 ^A,b^	307.67 ± 9.23 ^B,b^	486.89 ± 14.61 ^B,b^	280.49 ± 8.41 ^b^
	large intestine	0.14 ± 0.00 ^A,c^	0.46 ± 0.01 ^A,c^	1.86 ± 0.06 ^B,c^	1.68 ± 0.05 ^B,c^	1.45 ± 0.05 ^c^
**galic acid**	gastric	0.79 ± 0.02 ^A,a^	8.35 ± 0.25 ^A,a^	4.98 ± 0.15 ^B,a^	5.92 ± 0.28 ^B,a^	0.65 ± 0.02 ^a^
	small intestine	47.23 ± 1.42 ^A,b^	10.47 ± 0.41 ^A,b^	78.71 ± 2.36 ^B,b^	66.03 ± 1.98 ^B,b^	110.14 ± 3.30 ^b^
	large intestine	0.15 ± 0.00 ^A,c^	0.82 ± 0.05 ^A,c^	2.05 ± 0.09 ^B,c^	0.65 ± 0.02 ^B,c^	7.01 ± 0.21 ^c^
**3,4-dihydroxybenzoic acid**	gastric	0.55 ± 0.00 ^A,a^	4.09 ± 0.12 ^A,a^	0.85 ± 0.03 ^B,a^	2.96 ± 0.09 ^B,a^	0.72 ± 0.02 ^a^
	small intestine	12.14 ± 0.36 ^A,b^	0.92 ± 0.03 ^A,b^	114.37 ± 3.43 ^B,b^	45.14 ± 1.35 ^B,b^	49.79 ± 1.49 ^b^
	large intestine	0.00 ± 0.00 ^A,a^	0.01 ± 0.02 ^A,c^	19.04 ± 0.57 ^B,c^	11.32 ± 0.34 ^B,c^	80.04 ± 2.40 ^c^
**4-hydroxybenzoic acid**	gastric	0.00 ± 0.00 ^A,a^	3.47 ± 0.10 ^A,a^	0.32 ± 0.01 ^B,a^	0.00 ± 0.00 ^B,a^	1.34 ± 0.04 ^a^
	small intestine	0.02 ± 0.00 ^A,a^	0.01 ± 0.00 ^A,b^	5.03 ± 0.15 ^B,b^	11.82 ± 0.35 ^B,b^	7.93 ± 0.24 ^b^
	large intestine	0.09 ± 0.02 ^A,a^	0.03 ± 0.01 ^A,b^	0.16 ± 0.06 ^A,c^	0.06 ± 0.02 ^A,c^	0.09 ± 0.00 ^c^
**3-hydroxybenzoic acid**	gastric	0.00 ± 0.02 ^A,a^	0.00 ± 0.00 ^A,a^	0.02 ± 0.01 ^A,a^	0.02 ± 0.01 ^B,a^	0.01 ± 0.00 ^a^
	small intestine	0.24 ± 0.01 ^A,b^	3.98 ± 0.12 ^A,b^	1.28 ± 0.04 ^B,b^	5.73 ± 0.17 ^B,b^	0.66 ± 0.02 ^b^
	large intestine	0.01 ± 0.00 ^A,a^	0.32 ± 0.02 ^A,a^	0.72 ± 0.02 ^B,c^	7.05 ± 0.21 ^B,c^	4.96 ± 0.15 ^c^
**elagic acid**	gastric	2.00 ± 0.12 ^A,a^	3.49 ± 0.10 ^A,a^	1.24 ± 0.04 ^B,a^	3.16 ± 0.09 ^B,a^	3.38 ± 0.10 ^a^
	small intestine	0.90 ± 0.03 ^A,b^	0.18 ± 0.01 ^A,b^	19.82 ± 0.59 ^B,b^	56.86 ± 1.71 ^B,b^	26.59 ± 0.80 ^b^
	large intestine	0.00 ± 0.00 ^A,a^	0.00 ± 0.00 ^A,c^	0.15 ± 0.00 ^B,c^	0.26 ± 0.01 ^B,c^	25.96 ± 0.78 ^b^
**(+)-catechin**	gastric	0.12 ± 0.00 ^A,a^	72.53 ± 2.18 ^A,a^	0.05 ± 0.03 ^B,a^	10.12 ± 0.30 ^B,a^	0.77 ± 0.02 ^a^
	small intestine	0.02 ± 0.00 ^A,b^	1.03 ± 0.03 ^A,b^	14.70 ± 0.44 ^B,b^	18.93 ± 0.57 ^B,b^	3.81 ± 0.11 ^b^
	large intestine	0.01 ± 0.00 ^A,c^	0.03 ± 0.00 ^A,c^	0.02 ± 0.00 ^B,a^	0.18 ± 0.01 ^B,c^	1.57 ± 0.05 ^c^
**procyanidin B2**	gastric	0.14 ± 0.00 ^A,a^	0.94 ± 0.03 ^A,a^	0.56 ± 0.02 ^B,a^	1.98 ± 0.06 ^B,a^	2.54 ± 0.08 ^a^
	small intestine	0.00 ± 0.01 ^A,b^	0.00 ± 0.00 ^A,b^	1.84 ± 0.06 ^B,b^	1.80 ± 0.05 ^B,b^	4.76 ± 0.14 ^b^
	large intestine	0.00 ± 0.00 ^A,b^	0.00 ± 0.00 ^A,b^	0.94 ± 0.03 ^B,c^	0.52 ± 0.04 ^B,c^	4.29 ± 0.13 ^c^
**(-)-epicatechin**	gastric	30.52 ± 0.92 ^A,a^	181.99 ± 5.46 ^A,a^	0.38 ± 0.01 ^B,a^	6.65 ± 0.20 ^B,a^	2.20 ± 0.07 ^a^
	small intestine	0.00 ± 0.03 ^A,b^	0.00 ± 0.01 ^A,b^	264.30 ± 7.93 ^B,b^	186.00 ± 5.58 ^B,b^	72.34 ± 2.17 ^b^
	large intestine	0.00 ± 0.01 ^A,b^	0.01 ± 0.00 ^A,b^	9.57 ± 0.29 ^B,c^	6.86 ± 0.21 ^B,a^	18.63 ± 0.56 ^c^
**quercetin-3-glucoside**	gastric	0.00 ± 0.01 ^A,a^	0.01 ± 0.00 ^A,a^	0.00 ± 0.00 ^A,a^	0.00 ± 0.01 ^A,a^	0.00 ± 0.00 ^a^
	small intestine	1.01 ± 0.03 ^A,b^	0.00 ± 0.01 ^A,a,b^	1.67 ± 0.05 ^B,b^	3.81 ± 0.11 ^B,b^	2.30 ± 0.07 ^b^
	large intestine	0.04 ± 0.02 ^A,c^	0.00 ± 0.00 ^A,b^	0.00 ± 0.00 ^B,a^	0.00 ± 0.00 ^A,a^	0.00 ± 0.00 ^a^
**quercetin-3-galactoside**	gastric	0.03 ± 0.00 ^A,a^	0.09 ± 0.01 ^A,a^	0.10 ± 0.01 ^B,a^	0.20 ± 0.01 ^B,a^	0.09 ± 0.00 ^a^
	small intestine	0.13 ± 0.02 ^A,b^	0.05 ± 0.00 ^A,b^	0.98 ± 0.03 ^B,b^	1.37 ± 0.04 ^B,b^	1.10 ± 0.03 ^b^
	large intestine	0.02 ± 0.00 ^A,c^	0.13 ± 0.00 ^A,c^	1.20 ± 0.04 ^B,c^	1.13 ± 0.03 ^B,c^	2.81 ± 0.08 ^c^
**Total**	gastric	35.07 ± 1.12 ^A,a^	329.24 ±9.88 ^A,a^	13.52 ± 0.41 ^B,a^	162.93 ± 4.89 ^B,a^	22.93 ± 0.69 ^a^
	small intestine	84.84 ± 2.54 ^A,b^	53.50 ± 1.61 ^A,b^	835.58 ± 25.07 ^B,b^	932.16 ± 27.96 ^B,b^	654.50 ± 19.64 ^b^
	large intestine	0.74 ± 0.06 ^A,c^	1.87 ± 0.08 ^A,c^	37.72 ± 1.13 ^B,c^	31.87 ± 0.96 ^B,c^	176.60 ± 5.30 ^c^

RJ-royal jelly; HH-honeydew honey; WEAX-water extractable arabinoxylans. Uppercase letters followed by different letters indicate significant differences in individual phenolic content in the microcapsules obtained after spray drying bee products with WEAX depending on the type of the core material used for their preparation (*n* = 3; *p* ≤ 0.05); lowercase letters followed by different letters indicate significant differences in particular phenolic content in the individual sample depending on the digestive fraction (*n* = 3; *p* ≤ 0.05); data are presented as mean ± SD.
